# The Complete Chloroplast Genomes of *Punica granatum* and a Comparison with Other Species in Lythraceae

**DOI:** 10.3390/ijms20122886

**Published:** 2019-06-13

**Authors:** Ming Yan, Xueqing Zhao, Jianqing Zhou, Yan Huo, Yu Ding, Zhaohe Yuan

**Affiliations:** 1 Co-Innovation Center for Sustainable Forestry in Southern China, Nanjing Forestry University, Nanjing 210037, China; punica24@njfu.edu.cn (M.Y.); zhaoxq402@gmail.com (X.Z.); wangying@njfu.edu.cn (J.Z.); xinhuizhang888@gmail.com (Y.H.); punica2421@gmail.com (Y.D.); 2 College of Forestry, Nanjing Forestry University, Nanjing 210037, China; 3 College of Landscape Architecture, Nanjing Forestry University, Nanjing 210037, China

**Keywords:** pomegranate, chloroplast genome, sequence diversity, site-specific selection, phylogeny

## Abstract

Pomegranates (*Punica granatum* L.) are one of the most popular fruit trees cultivated in arid and semi-arid tropics and subtropics. In this study, we determined and characterized three complete chloroplast (cp) genomes of *P. granatum* cultivars with different phenotypes using the genome skimming approach. The complete cp genomes of three pomegranate cultivars displayed the typical quadripartite structure of angiosperms, and their length ranged from 156,638 to 156,639 bp. They encoded 113 unique genes and 17 are duplicated in the inverted regions. We analyzed the sequence diversity of pomegranate cp genomes coupled with two previous reports. The results showed that the sequence diversity is extremely low and no informative sites were detected, which suggests that cp genome sequences may be not be suitable for investigating the genetic diversity of pomegranate genotypes. Further, we analyzed the codon usage pattern and identified the potential RNA editing sites. A comparative cp genome analysis with other species within Lythraceae revealed that the gene content and organization are highly conserved. Based on a site-specific model, 11 genes with positively selected sites were detected, and most of them were photosynthesis-related genes and genetic system-related genes. Together with previously released cp genomes of the order Myrtales, we determined the taxonomic position of *P. granatum* based on the complete chloroplast genomes. Phylogenetic analysis suggested that *P. granatum* form a single clade with other species from Lythraceae with a high support value. The complete cp genomes provides valuable information for understanding the phylogenetic position of *P. gramatum* in the order Myrtales.

## 1. Introduction

Pomegranates (*Punica granatum* L.) are an economically important fruit tree of the tropical and subtropical regions of the world. It is native to central Asia and has been highly praised in many human cultures since ancient times [1]. Pomegranates have showy edible fruit with a high content of anthocyanins and flavonoids [2,3]. It has been well demonstrated that pomegranates are valuable to human health due to high levels of flavonoids and anthocyanins, which are considered potent antioxidants offering protection against heart disease and cancer [4,5]. Also, the pomegranate tree is suitable for genetic analysis due to its short juvenile period, and the high number of progenies [6]. As important resources for basic research and crop improvement, the genome of *P. granatum* ‘Taishanhong’ has been determined [7]. This genome will shed new light on the understanding of some unique biological processes and pomegranate breeding. Compared to the nuclear genome, the complete chloroplast genome is a low-cost and efficient way to get valuable genomic resources that can be used to understand evolution at multiple taxonomic levels [8,9] and analyze the population [10] because of its highly conserved structures and comparatively moderate substitution rates [11].

Chloroplasts (cp) are the photosynthetic organelles of the plant cells, which are derived from free-living cyanobacteria through endosymbiosis [12]. Apart from playing key roles in photosynthesis, chloroplasts are also responsible for other aspects of plant physiology and development [13]. A new study found that chloroplast retrograde signaling can regulate nuclear alternative splicing of a subset of *Arabidopsis thaliana* transcripts [14,15]. Interestingly, researchers have found that chloroplasts play diverse roles in plant defense, including contributing to the production of defense compounds [16]. Chloroplasts contain their own genome, the chloroplast DNA (cpDNA), which is highly conserved in genomic structure, gene content, and gene order. Cp genomes have been proved to be an effective biological tool for rapid and accurate species recognition as super-barcode [17,18]. With the advent of high-throughput sequencing technology, the increasing number of cp genomes of fruit crops has been published [19,20,21]. Before the development of next-generation sequencing technology, cp genome assembly was usually based on conventional primer walking strategies [22,23], which are laborious and costly. It is now convenient to obtain complete a cp genome by using genomic DNA extracted from the leaf tissue, because a large number of cp genomes are present in the sample [24]. Based on homology to cp from related species, these reads from cp can be assembled into circle genomes. Many bioinformatic tools have been developed to recover the cp genome sequence from total genomic DNA, such as NOVOPlasty [25], chloroExtractor [26], and GetOrganelle [27].

In the present study, we obtained cp genomes of pomegranates from three phenotypically different cultivars using the whole genome sequencing data. This study aimed to conduct a comprehensive analysis of the pomegranate cp genome, including gene content, genomic structure, codon usage, and potential RNA editing sites. In addition, combined with previously published cp genomes of Myrtales, phylogenetic analysis was performed to determine the taxonomic position of *P. granatum*. The results obtained here will provide valuable information for understanding the phylogenetic position of pomegranates and the evolutionary history of the order Myrtales.

## 2. Results and Discussion

### 2.1. General Features of Pomegranate Chloroplast Genomes

**Figure 1:**
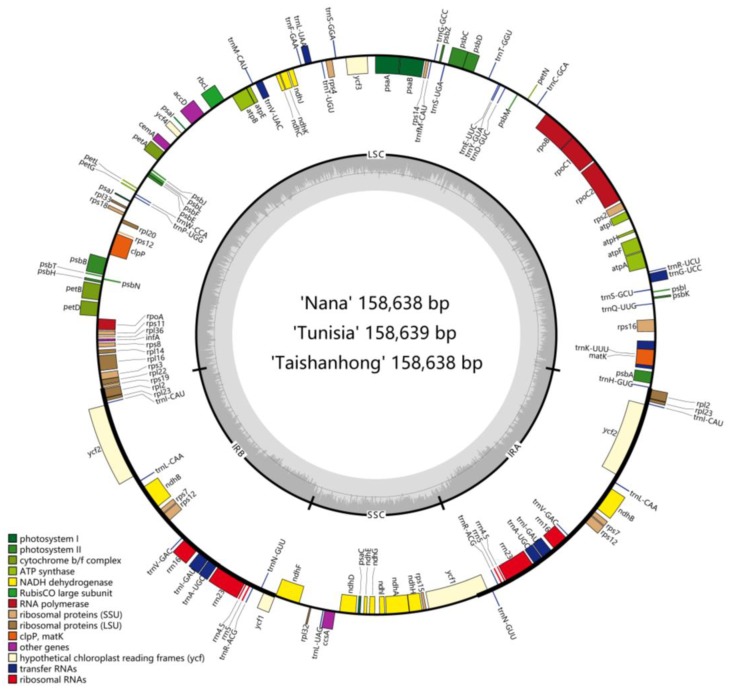
Chloroplast genome maps of *P. granatum*. Genes drawn outside the outer circle are transcribed clockwise, and those inside are transcribed counter-clockwise. Genes belonging to different functional groups are color-coded.

The complete cp genomes of ‘Nana’, ‘Tunisia’, and ‘Taishanhong’ were de novo assembled using whole genome sequencing data with GetOrganelle [27]. The cp genomes of ‘Nana’, ‘Tunisia’, and ‘Taishanhong’ were found to be 158,638, 158,639, and 158,638 bp in size, respectively. All of them exhibited a typical quadripartite structure, consisting of a pair of IRs separated by a large single copy region (LSC) and a small single copy region (SSC) (Figure 1). There are identical sets of 113 genes with the same gene order, including 79 protein-coding, 30 tRNA, and 4 rRNA genes. Six protein-coding genes (*rps7*, *rps12*, *rpl2*, *rpl23*, *ndhB*, *ycf2*), seven tRNA genes (*trnI-CAU*, *trnN-GUU*, *trnR-ACG*, *trnA-UGC*, *trnI-GAU*, *trnV-GAC*, *trnL-CAA*), and all rRNA genes (4.5S, 5S, 16S, 23S) are located at the IR regions. Eleven of the protein-coding genes and six of the tRNA genes contain introns, 14 of which contain a single intron, whereas three (*rps12*, *ycf3*, *clpP*) have two introns (Table 1). In particular, the *rps12* is a trans-spliced gene, whose first exon is located in the LSC, while the second and third exons reside in IRs. The *infA* gene was identified as a pseudogene because of the accumulation of the premature stop codons [28]. Another pseudogene *ycf1* existed because of the incomplete duplication of the normal copy of *ycf1* in the IRa and SSC junction, which is identical with previous reports [29,30]. There are some exceptions where non-ATG codons were identified as start codons, such as ACG for *psbL*, GTG for *rps19*, and ACG for *ndhD*. Alternate start codons have been found in other plant species [31]. Alternate start codons are still translated as Met when they are the start of a protein because a separate transfer RNA is used for initiation [32]. The overall GC content was 36.92%; this was consistent with previously reported GC content of IRs (42.78%) being higher than that of the LSC (34.89%) and SSC (30.64%) [33]. The high GC percentage of IRs could be due to the presence of rRNA sequences in these regions [34].

Chloroplast DNA has already been used in accessing the genetic diversity and phylogenetic structure at an intraspecies level. For instance, hypervariable regions of cp DNA such as *atpB-rbcL*, *trnL-trnF* and *rps16-trnQ* were used to assess the genetic diversity of Tunisian apricot accessions [35]. Also, chloroplast microsatellite loci were used to investigate the genetic diversity of Iranian pomegranate genotypes [36]. In our present study, the sequence diversity of pomegranate cp genomes was investigated combined with two previously reported cp genomes (NC_035240, MG878386). The results showed that the sequence diversity of pomegranates is extremely low (0.0008). Only 42 singleton variable sites were detected, and there were no parsimony variable sites in the alignment of the cp genomes of the five pomegranate accessions. Therefore, we propose that cp genome sequences might not be appropriate for investigating the genetic diversity of pomegranate genotypes.

### 2.2. Codon Usage Bias

**Figure 2:**
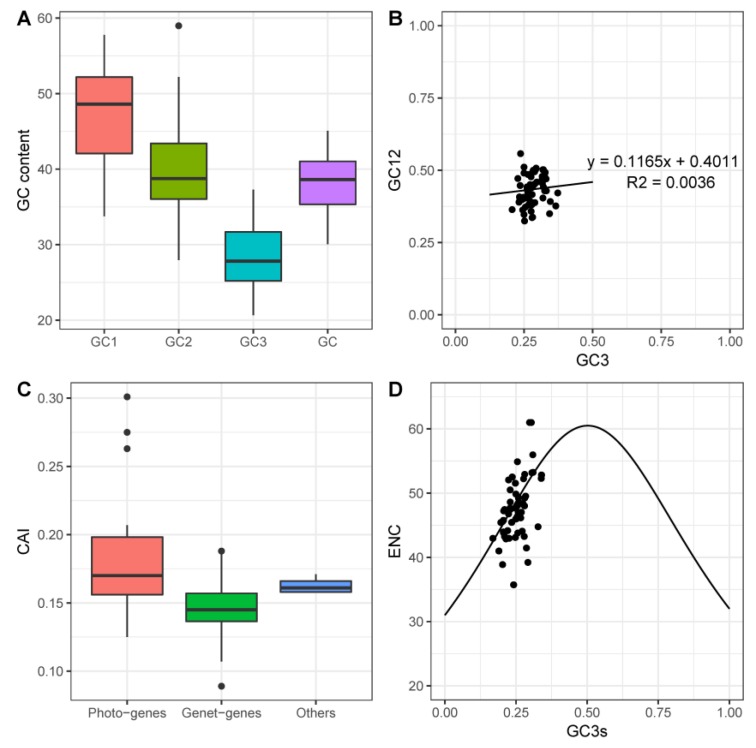
The codon usage pattern of the pomegranate chloroplast (cp) genome. (**A**) GC content on three different positions. (**B**) Neutrality plot (GC12 against GC3). (**C**) The codon adaptation index (CAI) value of gene sets with different functions. (**D**) Relationship between GC3 and effective number of codons (ENC) (ENC-plot). The expected ENC from GC3 is shown as a solid.

As an essential evolutionary feature, the codon usage pattern has been widely investigated in many plant species [37,38,39]. In our study, we explored the codon usage pattern in the cp genomes of pomegranates. Protein-coding genes with more than 300 nucleotides were selected for further analysis. Firstly, the base composition on three different codon positions was determined, and the data are displayed in Figure 2A. The results indicated that the average GC content of the first (GC1), second (GC), and third codon positions (GC3) were 47.04, 39.79, and 28.34%, respectively. The base compositions of the three different positions were distributed unevenly. The average GC3 content was significantly lower than those of GC1 and GC2. The results of neutrality plots (Figure 2B) showed that no significant correlation (R2 = 0.0036) between GC12 and GC3 was observed, which suggests that selective pressure affects the codon usage bias in the pomegranate cp genomes [40,41]. The codon adaptation index (CAI) value (Figure 2C) ranged from 0.5 to 1 with a default *E. coli* reference gene set as the reference. According to their functions in the chloroplast, the protein-coding genes can be classified into three categories: photosynthesis related genes (photo-genes), genetic system related genes (genet-genes), and other genes. A recent study about codon usage bias of cp genomes in cultivated and wild Solanum species concluded that photo-genes always had higher CAI values than genet-genes because the expression level of photo-genes is relatively higher than that of genet-genes [42]. The same result was also observed in the cp genome of pomegranates. The main reason is probably due to the fact that photo-genes may have a higher codon usage bias for the requirement of high gene expression than do the genet-genes in the plant cp genomes. Furthermore, the relationship between bas compositions and codon usage was investigated by ENC-plot (Figure 2D). Effective number of codons (ENC) values ranged from 35.73 to 61, suggesting that codon usage bias is relatively weak in the pomegranate cp genomes. The distribution of most genes was far away from the standard curve, which shows that there are other factors that affect the codon usage, other than base compositions [43,44,45]. As the cp genomes were highly AT-rich, it was not surprising that AT-ending codons would be predominant in the protein-coding genes. The results are also consistent with the mutational bias towards AT being the force driving the strong bias of codon usage of plan cp genomes [43]. Also, the Arg amino acid coded with the AGA codon was the most frequent codon with a relative synonymous codon usage (RSCU) value 2.02 (Table 2).

### 2.3. RNA Editing Sites

RNA editing is a posttranscriptional process, which has been experimentally identified in organellar transcriptomes from several species [46,47]. It mainly involves the conversion of cytidine to uridine, which generally results in amino acid changes. Therefore, knowing where sites of RNA editing exist in the organelle transcriptome could provide information for understanding the structure and function of the translated proteins [48]. The potential RNA editing sites in the pomegranate cp genome were predicted using the online program PREP. A total of 64 editing sites in 20 protein-coding genes were identified (Table 3). The *ndhB* gene had the highest number of potential editing sites (11), followed by the *ndhD* gene (9). In accordance with previous reports [49,50], we observed that most conversions at the codon positions changed from serine (S) to leucine (L) and most RNA editing sites led to amino acid changes from polar to apolar, which resulted in an increase in protein hydrophobicity.

### 2.4. Sequence Diversity of the Chloroplast Genomes among Lythraceae Species

**Figure 3:**
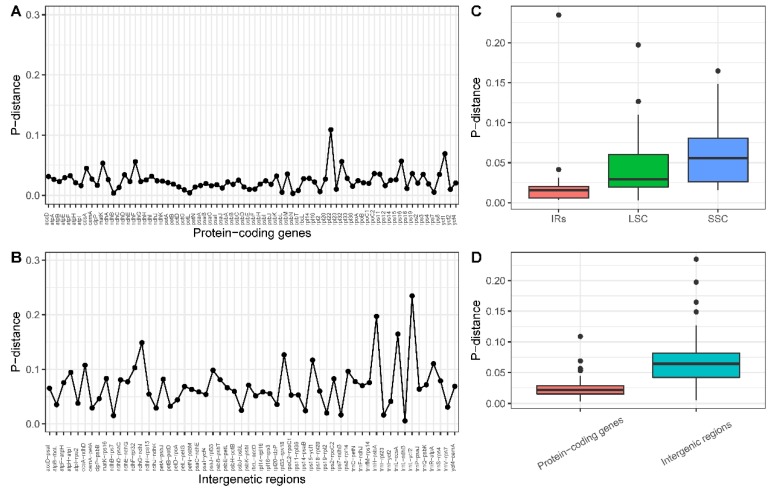
The genetic distance based on Kimura’s two-parameter model. (**A**) The P-distance value of protein-coding genes. (**B**) The P-distance value of intergenic regions. (**C**) Boxplots of P-distance value difference among LSC, SSC, and IRs. (**D**) Boxplots of P-distance value differences between protein-coding genes and intergenic regions.

Four complete cp genomes within Lythraceae, available in GenBank with our newly assembled ‘Taishanhong’, were selected to analyze the sequence diversity. The mean P-distance value was designated to represent the level of divergence. The genetic distance of all 76 protein-coding genes (Figure 3A) ranged from 0.003053 (*psbN*) to 0.108932 (*rpl22*), with an average of 0.024379. The intergenic regions had a relatively higher genetic distance (Figure 3B) compared to the protein-coding regions, ranging from 0.005621 (*trnL-ndhB*) to 0.23463 (*trnL-ycf2*) with an average value of 0.069775. The results agree with previous reports that intergenic regions showed greater divergence than coding regions (Figure 3D) [51]. The SSC region exhibited higher divergence levels than the LSC and IRs (Figure 3C). Three intergenic regions with genetic distance values over the 95th percentile were considered as highly divergent regions, including *trnH-psbA*, *trnL-ccsA*, and *trnL-ycf2*. These highly variable regions may be regarded as potential molecular markers for application in phylogenetic analyses in Lythraceae.

### 2.5. Structure Comparison among the Chloroplast Genomes of Lythraceae Species

**Figure 4:**
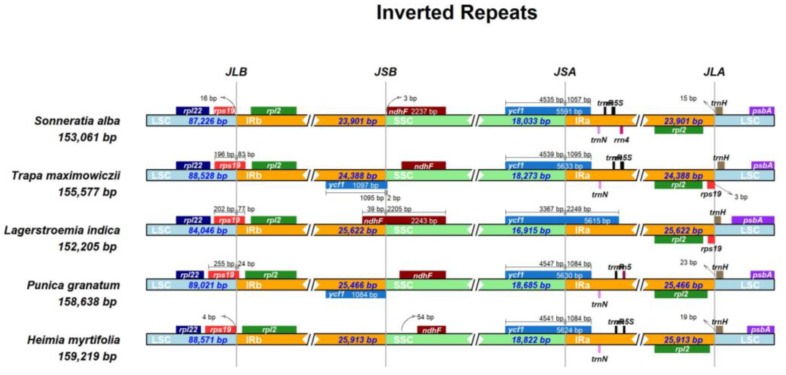
Comparison of the borders of the LSC, SSC, and IRs regions among five Lythraceae cp genomes.

Five complete cp genomes within Lythraceae were selected for comparison with each other. The genome sizes was ranged from 152,205 to 159,219 bp. The length of the LSC, SSC, and IRs varied in the range of 84,046–89,021 bp, 16,914–18,821 bp, and 23,902–25,914 bp, respectively. *Lagerstroemia indica* has the smallest genome, and this difference is mostly attributed to variation in the length of the LSC and IR regions (Table 4). A detailed comparison on four borders between the two IRs and the two single-copy regions showed that the border structures were highly similar with one another (Figure 4). However, a slight difference in junction positions was observed among these five cp genomes. For instance, the *ndhF* gene was located at the SSC region in *Sonneratia alba*, *Trapa maximowiczii*, *Punica granatum*, and *Heimia myrtifolia*, while it varied from 3 to 54 bp apart from the IRb/SSC junction. However, the *ndhF* gene crossed over the IRb/SSC region in *Lagerstroemia indica*. The *rps19* gene was located in the junction of the LSC/IRb in *Trapa maximowiczii*, *Lagerstroemia indica*, and *Punica granatum*, with 24–83 bp located in the IRb. However, in *Heimia myrtifolia* and *Sonneratia alba*, the *rps19* gene was fully located in the LSC region, and 4–16 bp apart from the LSC/IRb border. Overall, the IR boundary regions varied slightly in the Lythraceae cp genomes. IR expansion and contraction often results in genome size variations among various plant lineages, which can be used to study the phylogenetic classification and the genome evolution among plant lineages. Three reasons may explain the diversification of the IR boundary region sequences: the first is intramolecular recombination, the second is the presence of multiple repeat sequences, and the third is the indels, which caused a mismatch that resulted in the upstream sequence becoming a single copy [52].

**Figure 5:**
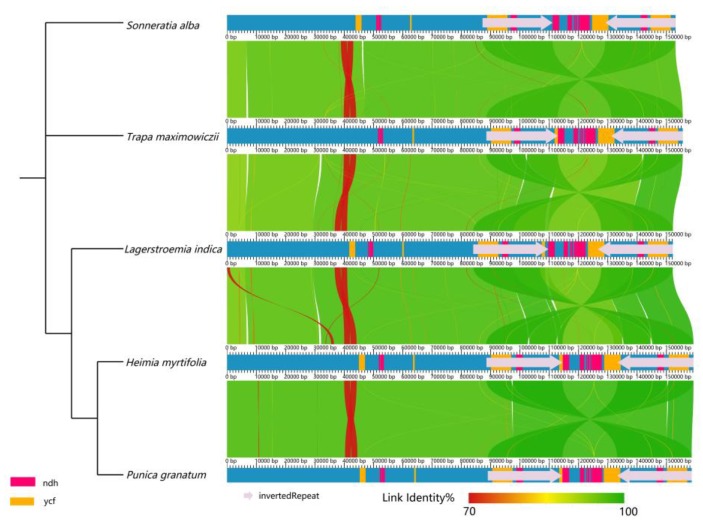
Co-linear analysis of five cp genomes within Lythraceae.

Pairwise alignment of the *P. granatum* cp genome with the other Lythraceae species revealed a high degree of synteny and gene order conservation (Figure 5), suggesting an evolutionary conservation of the Lythraceae cp genomes at the genome-scale level.

### 2.6. Positive Selection Analysis

The ratios of non-synonymous (dN) and synonymous (dS) substitutions for 75 protein-coding genes among five Lythraceae were calculated based on the site-specific model. Eleven genes with positively selected sites within the Lythraceae family were identified (Table 5). Those genes contained one subunit of acetyl-CoA carboxylase (*accD*), one photosystem I subunit gene (*psaI*), two NADH-dehydrogenase subunit genes (*ndhF*, *ndhJ*), one ribosome large subunit gene (*rpl22*), five ribosome small subunit genes (*rps2*, *rps4*, *rps7*, *rps8*, *rps12*), and the *ycf1* gene. According to the M8 model, the ycf1 gene possessed 10 positive sites, followed by *ndhF* (7) and *rpl22* (5). The other eight genes each had only one positive site. The Photo-genes included four genes (*accD*, *psaI*, *ndhF*, *ndhJ*). The Genet-genes included six genes (*rpl22*, *rps2*, *rps4*, *rps7*, *rps8*, *rps12*). The *ycf1* gene was considered as the other gene. Most positively selected genes were genetic system or photosynthesis related genes, which indicated that the chloroplast functional genes played vital roles during the plant evolution [53,54].

### 2.7. The Phylogenetic Position of P. granatum

Complete cp genomes are important as they can provide information for understanding the phylogenetic relationships at multiple taxonomic levels. For example, recent phylogenetic analyses based on protein-coding genes of the cp genome provided the broad phylogenetic framework for Viridiplantae, which has significant value both to evolutionary biologists and ecologists [55]. The genus *Punica* belongs to the order Myrtales and most likely originated from Saxifragales. However, the placement of genus *Punica* under different families such as Punicaceae, Lythraceae, and Myrtaceae has remained debatable [6]. Recent phylogenomic analysis based on 106 single-copy nuclear genes was performed and supported that *P. granatum* belongs to the Lythraceae family rather than the monogeneric Punicaeceae family [7].

To determine the position of *P. granatum* and to further analyze the relationships within Myrtales, we used the complete cp genome sequences to perform the phylogenetic analysis. Eighty-five species representing five families from the order Myrtales were selected. Two species from the order Vitales (*Vitis acerifolia*, *Vitis vinifera*) were selected as outgroups. To avoid systematic errors produced by poor alignment, we removed poorly aligned sites using Gblocks. After the removal of the ambiguously aligned regions, the alignment contained 106,571 sites in total, including 20,088 parsimony-informative sites, 9178 singleton sites, and 77,305 constant sites. The method of data analysis (ML and BI) did not affect the results, and the treetopologies of ML and BI were found to be the same. The phylogenetic relationships among five families were fully resolved, and node support values for the ML type were higher than 90.

**Figure 6:**
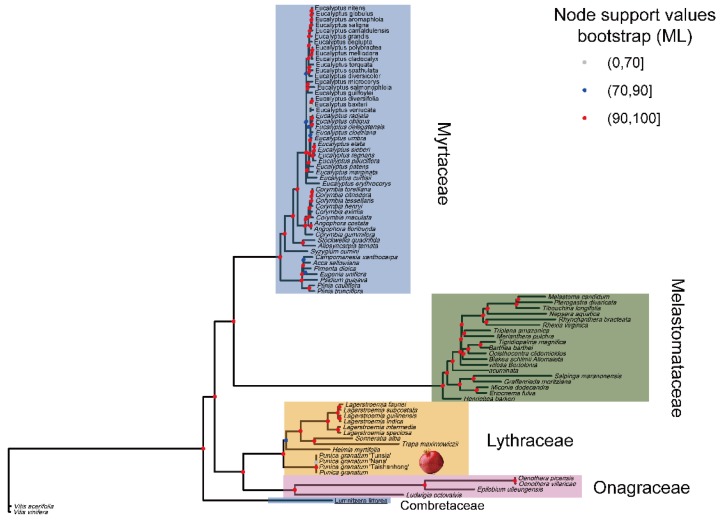
Phylogenetic tree was reconstructed using Maximum likelihood (ML) and Bayes inference (BI) methods based on complete cp genomes of the order Myrtales. Only the tree topology of the ML tree was presented.

The tree showed (Figure 6) that all the accessions of the pomegranate were grouped into a single clade with other closely related species of the Lythraceae family. The monophyly of the family Lythraceae and the sister relation with family Onagraceae is highly supported (>90). Myrtaceae were strongly supported as monophyletic and formed a sister relationship with Melastomataceae. Five pomegranate accessions formed a clade with zero or nearly zero branches length, which suggests that the cp genome might be of limited use for cultivar identification and population genetic studies of *P. granatum*. Our full genomic data set resolved the phylogenomic placement of *Punica* and provided strong support for most relationships of Myrtales.

## 3. Materials and Methods

### 3.1. Plant Material

Three *P. granatum* cultivars with distinct phenotypes were chosen to reconstruct the complete cp genome: ‘Nana’ is a dwarf pomegranate, which has a small and sour fruit with hard seeds. ‘Tunisia’ is a domesticated cultivar characterized as a normal-sized tree with sweet taste and soft seeds. ‘Taishanhong’ is a widely grown landrace in China, characterized as having a bright red peel with delicious taste and hard seeds. The materials used in this study were collected from the experimental orchard at Nanjing Forestry University. The voucher specimen was deposited in Nanjing Forestry University.

### 3.2. DNA Sequencing, Genome Assembly, and Annotation

Total genomic DNA was extracted from mature leaves using a modified CTAB protocol. Firstly, 1.0 μg Genomic DNA was sheared into an average fragment size of 350 bp by a Covaris S220 sonicator (Woburn, Massachusetts, MA, USA). Then, the size distribution and concentration of the libraries were determined using an Agilent 2100 Bioanalyzer and qualified by real-time PCR (2 nM), respectively. DNA libraries were sequenced on Illumina Hiseq X Ten (Nanjing, China) for at least 150 bp reads. The raw sequence data reported in this paper were deposited in the Genome Sequence Archive in Big Data Center [56], Beijing Institute of Genomics (BIG) [57], Chinese Academy of Sciences, under the BioProject with the accession number PRJCA001313. After the fragments were filtered and trimmed by the fastp program [58], clean reads were obtained. Subsequently, the high-quality paired-end reads were used to de novo assemble the complete cp genomes using the GetOrganelle program [27] with a combined k-mer of 95,105,125. Genome annotation was performed using the online program GeSeq [59] for the pomegranate cp genomes previously reported. The annotation results were inspected using Geneious [60] and adjusted manually as needed. The cp genome map was drawn using the online tool OGDRAW [61]. The complete cp genomes have been submitted to Genbank with accession number MK603511-MK603513.

### 3.3. Codon Usage

The complete cp genome of the pomegranate cultivar ‘Taishanhong’ was selected to analyze the codon usage pattern. The protein-coding genes with more than 300 nucleotides were extracted according to the annotation file. The GC content of GC1, GC2, and GC3 was calculated using an in-house python script. The codon usage indices were calculated by CodonW v1.4.4, including the relative synonymous codon usage (RSCU), codon adaptation index (CAI), and the effective number of codons (ENC). RSCU values were close to 1 indicating that all synonymous codons encoding the same amino acid were used equally. CAI is used to measure the extent of bias towards preferred codons in a gene. A higher CAI value means a stronger codon usage bias and a higher expression level. ENC is used to measure codon usage evenness. Its value ranges from 20 (extremely biased) to 61 (totally unbiased) [62].

### 3.4. RNA Editing Sites

Prediction of the possible RNA editing sites in *P. granatum* protein-coding genes were performed using the online program predictive RNA editor for plants (PREP) suite [63] with 35 genes as reference. Only those sites which had a cutoff value of 0.8 were kept.

### 3.5. Sequence Diversity

Four cp genomes from Lythraceae were downloaded from GenBank, including *Lagerstroemia indica* (NC_030484), *Heimia myrtifolia* (MG921615), *Sonneratia alba* (NC_039975), and *Trapa maximowiczii* (NC_037023). These four cp genomes together with that of our newly assembled ‘Taishanhong’ genome were used to detect the divergent hot spot. Intergenic and protein-coding regions from five Lythraceae cp genomes were extracted using an in-house python script. Multiple sequence alignment was performed using MAFFT [64] and the mean P-distances were calculated using R package ‘ape’ [65] with Kimura’s two-parameter model.

### 3.6. Structure Comparison

IR expansion and contraction of cp genomes among the five Lythraceae species mentioned above were analyzed using IRscope (Helsinki, Finland) [66]. We also conducted a co-linear analysis. A pairwise alignment was achieved by the lastz program. The results were visualized using AliTV (Wurzburg, Germany) [67].

### 3.7. Positive Selection Analysis

In order to detect the protein-coding genes under selection in Lythraceae, the sequences for each gene were aligned separately using the Muscle (codon) implemented in MEGA [68], and the Maximum likelihood phylogenetic tree based on complete cp genome sequences was constructed using IQ-tree [69]. The site-specific model was performed to test for natural selection using the CODEML algorithm [70] implemented in EasyCodeML [71]. Six codon substitution models described as M0, M1a, M2a, M3, M7, and M8 were investigated. This model allowed the ω ratio to vary among sites with a fixed ω ratio in all branches in order to test for site-specific evolution in the gene phylogeny. Two likelihood ratio tests were performed to detect positively selected sites: M1a (neutral) vs. M2a (positive selection), M7 (β) vs. M8 (β and ω), and M0 (one-ratio) vs. M3 (discrete), which were compared using a site-specific model [72].

### 3.8. Phylogenetic Analysis

To determine the phylogenetic position of *P. granatum*, phylogenetic analysis was performed using the complete cp genomes in the Myrtales. The cp genomes previously published in the Myrtales and two species from Vitales were downloaded from NCBI using an in-house python script. Multiple sequences alignment was achieved by HomBlocks pipelines [73]. Some poorly aligned regions were removed with Gblocks [74]. Two methods were employed to construct a phylogenetic tree, including Maximum likelihood (ML), and Bayes inference (BI). The dataset was unpartitioned. ML was implemented in IQ-tree [69] under the best-fit model selected by using ModelFinder [75] according to Akaike information criterion (AIC). The ML tree was inferred with 1000 bootstrap replicates (the ‘-bb’ options). BI was performed with MrBayes [76] under the best-fit model determined by Modeltest with the AIC. The Marjkov chain Monte Carlo (MCMC) analysis was run for 2 × 200,000 generations. Trees were sampled at every 1,000 generations with the first 25% discarded as burn-in. The stationarity was considered to be reached when the average standard deviation of split frequencies remained below 0.001. Phylogenetic trees with bootstrap values (BS) and posterior probabilities (PP) were visualized using R package ‘ggtree’ [77].

## 4. Conclusions

Next generation whole genome shotgun sequences of plant species often contain numerous reads that are derived from the cp genomes, which provides a unique opportunity to assemble complete cp genomes. This method of using low coverage of the whole genome sequencing data to recover highly repetitive genome regions such as organelle genomes is called the “whole genome skimming approach”. In the present study, we determined and characterized the complete cp genomes of three *P. granatum* cultivars using the whole genome sequencing data. Sequence diversity analysis revealed that cp genome sequences may not be suitable for investigating the genetic diversity of pomegranate genotypes. The genome sequencing data of three different cultivars are valuable resources for pomegranate breeding programs. The complete cp genome sequences that were newly assembled in our study could provide valuable information for understanding the evolutionary relationships among the Myrtales.

## Figures and Tables

**Table 1:** The groups of genes within the *P. granatum* chloroplast genome.

Group of Genes	Gene Names
Photosystem I	*psaA, psaB, psaC, psaI, psaJ*
Photosystem II	*psbA, psbB, psbC, psbD, psbE, psbF, psbH, psbI, psbJ, psbK, psbL, psbM, psbN, psbT, psbZ*
Cytochrome b/f complex	*petA, petB^a^, petD^a^, petG, petL, petN*
ATP synthase	*atpA, atpB, atpE, atpF^a^, atpH, atpI*
NADP dehydrogenase	ndhA^a^, ndhB^*a^, ndhC, ndhD, ndhE, ndhF, ndhG, ndhH, ndhI, ndhJ, ndhK
RubisCO large subunit	*rbcL*
RNA polymerase	*rpoA, rpoB, rpoC1^a^, rpoC2*
Ribosomal proteins (SSU)	rps2, rps3, rps4, rps7*, rps8, rps11, rps12*^b^, rps14, rps15, rps16^a^, rps18, rps19
Ribosomal proteins (LSU)	rpl2^*^, rpl14, rpl16^a^, rpl20, rpl22, rpl23*, rpl32, rpl33, rpl36
Hypothetical chloroplast reading frames	ycf1, ycf2*, ycf3^b^, ycf4
Translation initiation factor IF-1	*infA*
Acetyl-CoA carboxylase	*accD*
Cytochrome c biogenesis Maturase	*matK*
ATP-dependent protease	*clpP^b^*
Inner membrane protein	*cemA*
Ribosomal RNAs	rna4.5S*, rna5S*, rna16S^*^, rna23S*
Transfer RNAs	trnA-UGC*^a^, trnC-GCA, trnD-GUC, trnE-UUC, trnF-GAA, trnfM-CAU, trnG-GCC, trnG-UCC^a^, trnH-GUG, trnI-CAU*^a^, trnI-GAU*, trnK-UUU^a^, trnL-CAA*, trnL-UAA^a^, trnL-UAG, trnM-CAU, trnN-GUU*, trnP-UGG, trnQ-UUG, trnR-ACG*, trnR-UCU, trnS-GCU, trnS-GGA, trnS-UGA, trnT-GGU, trnT-UGU, trnV-GAC*, trnV-UAC^a^, trnW-CCA, trnY-GUA

One asterisk indicates that the genes contained two copies. a and b indicate one- and two-intron containing genes, respectively.

**Table 2:** Putative preferred codons in the *P. granatum* cp genome. RSCU = relative synonymous codon usage.

Amino Acid	Codon	Codon Frequency	RSCU	AA	Codon	Codon Frequency	RSCU
Phe	UUU*	4551	1.18	Ser	UCU*	2417	1.46
	UUC	3143	0.82		UCC	1577	0.96
Leu	UUA*	3112	1.41		UCA*	2278	1.38
	UUG*	2920	1.32		UCG	1268	0.77
	CUU*	2586	1.17	Pro	CCU*	1385	1.21
	CUC	1360	0.62		CCC	929	0.81
	CUA	1958	0.89		CCA*	1387	1.21
	CUG	1287	0.58		CCG	876	0.77
Ile	AUU*	4378	1.27	Thr	ACU*	1478	1.13
	AUC	2723	0.79		ACC	1095	0.84
	AUA	3246	0.94		ACA*	1759	1.35
Met	AUG	2760	1.00		ACG	886	0.68
Val	GUU*	2045	1.34	Ala	GCU*	1389	1.45
	GUC	1033	0.68		GCC	712	0.75
	GUA*	1891	1.24		GCA	1145	1.20
	GUG	1123	0.74		GCG	576	0.60
Tyr	UAU*	3606	1.37	Cys	UGU*	1410	1.17
	UAC	1665	0.63		UGC	993	0.83
TER	UAA*	2029	1.03	TER	UGA	2003	1.01
	UAG	1893	0.96	Trp	UGG	2392	1.00
His	CAU*	1908	1.38	Arg	CGU	888	0.7
	CAC	866	0.62		CGC	456	0.36
Gln	CAA*	2815	1.35		CGA*	1428	1.13
	CAG	1342	0.65		CGG	865	0.68
Asn	AAU*	3923	1.37	Ser	AGU	1441	0.87
	AAC	1800	0.63		AGC	923	0.56
Lys	AAA*	4768	1.31	Arg	AGA*	2560	2.02
	AAG	2538	0.69		AGG	1412	1.11
Asp	GAU*	2818	1.49	Gly	GGU	1642	1.01
	GAC	962	0.51		GGC	886	0.54
Glu	GAA*	3632	1.37		GGA*	2409	1.48
	GAG	1689	0.63		GGG	1569	0.96

Preferred codons (RSCU value > 1.0) are indicated with (*).

**Table 3:** Predicted RNA editing sites in the cp genome of *P. granatum.*

Gene	Nucleotide Position	Amino Acid Position	Codon Conversion	Score
*matK*	644	215	GCA (A) => GTA (V)	1
	1177	393	CGG (R) => TGG (W)	1
	1187	396	TCA (S) => TTA (L)	0.86
	1246	416	CAC (H) => TAC (Y)	1
*atpA*	791	264	CCC (P) => CTC (L)	1
*atpF*	92	31	CCA (P) => CTA (L)	0.86
*atpI*	23	8	ACC (T) => ATC (I)	1
*rps2*	422	141	TCG (S) => TTG (L)	1
*rpoC2*	3056	1019	GCA (A) => GTA (V)	0.86
	3998	1333	GCG (A) => GTG (V)	0.86
*rpoC1*	41	14	TCA (S) => TTA (L)	1
	1171	391	CCA (P) => TCA (S)	1
*rpoB*	338	113	TCT (S) => TTT (F)	1
	551	184	TCA (S) => TTA (L)	1
	566	189	TCG (S) => TTG (L)	1
	973	325	CTC (L) => TTC (F)	0.86
*rps14*	80	27	TCA (S) => TTA (L)	1
	149	50	TCA (S) => TTA (L)	1
*atpB*	1487	496	TCG (S) => TTG (L)	1
*accD*	794	265	TCG (S) => TTG (L)	0.8
	1403	468	CCT (P) => CTT (L)	1
*psbL*	2	1	ACG (T) => ATG (M)	1
*psbF*	77	26	TCT (S) => TTT (F)	1
*clpP*	559	187	CAT (H) => TAT (Y)	1
*ndhB*	28	10	CTC (L) => TTC (F)	1
	149	50	TCA (S) => TTA (L)	1
	467	156	CCA (P) => CTA (L)	0.8
	586	196	CAT (H) => TAT (Y)	1
	611	204	TCA (S) => TTA (L)	1
	737	246	CCA (P) => CTA (L)	1
	746	249	TCT (S) => TTT (F)	1
	830	277	TCA (S) => TTA (L)	1
	836	279	TCA (S) => TTA (L)	1
	1255	419	CAT (H) => TAT (Y)	1
	1481	494	CCA (P) => CTA (L)	1
*ndhF*	160	54	CTT (L) => TTT (F)	1
	586	196	CTT (L) => TTT (F)	0.8
*ccsA*	89	30	TCG (S) => TTG (L)	1
*ndhD*	2	1	ACG (T) => ATG (M)	1
	185	62	ACC (T) => ATC (I)	1
	313	105	CGG (R) => TGG (W)	0.8
	383	128	TCA (S) => TTA (L)	1
	674	225	TCG (S) => TTG (L)	1
	845	282	ACA (T) => ATA (I)	0.8
	878	239	TCA (S) => TTA (L)	1
	887	296	CCA (P) => CTA (L)	1
	1405	469	CTT (L) => TTT (F)	0.8
*ndhG*	155	52	CCA (P) => CTA (L)	1
	166	56	CAT (H) => TAT (Y)	0.8
	314	105	ACA (T) => ATA (I)	0.8
*ndhA*	341	114	TCA (S) => TTA (L)	1
	566	189	TCA (S) => TTA (L)	1
	1073	358	TCC (S) => TTC (F)	1

**Table 4:** Summary of the complete chloroplast genome characteristics of five species in Lythraceae.

Species	*Punica granatum*	*Lagerstromeia indica*	*Sonneratia alba*	*Trapa maximowicizz*	*Heimia myrtifolia*
Genome size	158,638	152,025	153,061	155,577	159,219
LSC size	89,021	84,046	87,226	88,528	88,571
SSC size	18,684	16,914	18,032	18,272	18,821
IR size	25,467	25,623	23,902	24,389	25,914
Number of genes	113	113	107	110	112
Protein-coding genes	79 (6)	79 (7)	79 (6)	77 (5)	78 (7)
tRNA genes	30 (7)	30 (7)	24 (5)	29 (9)	30 (6)
rRNA genes	4 (4)	4 (4)	4 (4)	4 (4)	4 (4)
Number of genes duplicated in IR	17	18	15	18	17
GC content	36.92	37.59	37.29	36.4	36.95
GenBank accession	MK603511	NC_030484	NC_039975	NC_037023	MG921615

**Table 5:** Log-likelihood values of the site-specific models, with detected sites having non-synonymous/synonymous (dN/dS) values > 1.

Gene Name	Models (Number of Parameters)	lnL	Likelihood Ratio Test *p*-Value	Positively Selected Sites
*accD*	M8 (12)	−2534.400824	0.0962956	125 G 0.955 *
	M7 (10)	−2536.741156		
*ndhF*	M8(12)	−4446.871610	0.000000002	292 N 0.961 *; 486 R 0.999 **; 487 I 0.975 *; 490 K 0.985 *; 518 N 0.969 *; 648 S 0.983 *; 738 F 0.995 **
	M7(10)	−4466.787914		
*ndhJ*	M8(12)	−806.644367	0.003291615	121 R 0.970 *
	M7(10)	−812.360744		
*psaI*	M8(12)	−139.985819	0.031547151	26 H 0.959*
	M7(10)	−143.4420		
*rpl22*	M8(12)	−942.833567	0.000497160	4 L 0.972 *; 5 Y 0.961 *; 73 P 0.962 *; 125 A 0.993 **; 126 R 0.994 **
	M7(10)	−950.440165		
*rps12*	M8(12)	−529.317591	0.002536102	117 K 0.974 *
	M7(10)	−535.294718		
*rps2*	M8(12)	−1171.788526	0.008240568	173 E 0.982 *
	M7(10)	−1176.587212		
*rps4*	M8(12)	−994.666749	0.007084882	28 P 0.959 *
	M7(10)	−999.616541		
*rps7*	M8(12)	−672.815064	0.000000001	84 T 1.000 **
	M7(10)	−693.433775		
*rps8*	M8(12)	−718.655799	0.001840922	59 L 0.989 *
	M7(10)	−724.953288		
*ycf1*	M8(12)	−11,993.590817	0.000001936	205 V 0.977 *; 206 F 0.975 *; 341 S 0.974 *; 495 S 0.952 *; 534 A 0.951 *; 1073 A 0.963 *; 1290 R 0.978 *; 1446 E 0.963 *; 1701 K 0.976 *; 1728 T 0.950 *
	M7(10)	−12,006.745724		

* *p* < 0.05; ** *p* < 0.01.
